# Skill Improvement Through Learning in Therapy (SKILT): A Study Protocol for a Randomized Trial Testing the Direct Effects of Cognitive Behavioral Therapy Skill Acquisition and Role of Learning Capacity in Depression

**DOI:** 10.32872/cpe.8475

**Published:** 2023-03-31

**Authors:** Sanne J. E. Bruijniks, Ulrike Frank, Brunna Tuschen-Caffier, Jessica Werthmann, Fritz Renner

**Affiliations:** 1 Department of Psychology, Clinical Psychology and Psychotherapy, University of Freiburg, Freiburg, Germany; 2 Department of Clinical Psychology, Utrecht University, Utrecht, the Netherlands; Philipps-University of Marburg, Marburg, Germany

**Keywords:** major depressive disorder (MDD), cognitive-behavioral therapy (CBT), cognitive behavioral therapy skills, mental imagery, experiment

## Abstract

**Background:**

To improve psychological treatments for major depressive disorder (MDD), a better understanding on how symptoms ameliorate during treatment is essential. In cognitive behavioral therapy (CBT), it is unclear whether procedures focused on the acquisition of CBT skills play a causal role in the improvement of CBT skills. In this randomized trial, we isolate a single CBT Skill Acquisition Procedure (CBTSAP) and test its direct effects on CBT skills and related therapy processes (i.e., change in (idiosyncratic) dysfunctional thinking and reward processing). We hypothesize that the CBTSAP causes improvements in CBT skills and related therapy processes compared to an active control condition. In addition, we hypothesize that individual differences in attentional bias and memory functioning (defined as learning capacity) moderate the effects of CBTSAP on outcomes and that using mental imagery as a cognitive support strategy to strengthen the effects of the CBTSAP will be most beneficial for patients with low learning capacity.

**Method:**

150 patients with MDD will be randomized to one of three conditions: 1. an active control condition, 2. CBTSAP, 2. CBTSAP plus mental imagery, all consisting of three sessions. Primary outcomes will be change in CBT skills, changes in (idiosyncratic) dysfunctional thoughts and behaviors, reward processing. Depressive symptoms are a secondary outcome. Measures of learning capacity will be conducted at baseline and tested as a potential moderator.

**Discussion:**

Knowing whether and for whom the acquisition of CBT skills leads to change in therapy processes and a subsequent reduction of depressive symptoms will inform on how to personalize and optimize psychotherapy outcomes for depression.

**Trial registration:**

The trial is registered at the German Clinical Trial Register (DKTR; registration number: DRKS00024116).

## Highlights

- Study protocol for a randomized trial to test direct effects of a procedure focused on CBT skill acquisition in MDD.
- Outcomes are CBT skills, dysfunctional thinking, reward processing and depressive symptoms.
- The role of learning capacity as a moderator will be investigated.
- Results will inform on the direct effects and individual differences in effects of CBT procedures.

## Background

Current psychological treatments for depression are only effective for half of the patients (Cuijpers et al., 2021). Response to psychological treatments is limited and relapse rates are high (Steinert et al., 2014; Verduijn et al., 2017; Vittengl et al., 2007). To improve and innovate psychological treatments, a better understanding of how symptoms improve during treatment is essential.

Psychotherapies aim to reduce depressive symptoms by mobilizing therapy processes that seem central to the development and maintenance of depressive symptoms. Therapy processes can be defined as the mechanisms inside the mind of the patient that are activated by the therapeutic procedures delivered by the therapist with the intent of producing change (Bruijniks et al., 2018). In cognitive behavioral therapy (CBT; Beck et al., 1979), one of the most investigated treatments for depression, therapeutic procedures focus on three major therapy processes: dysfunctional thinking, behavioral activation and the acquisition of CBT skills (Barber & DeRubeis, 1989; Lorenzo-Luaces et al., 2016). First, cognitive change procedures aim to change the process of dysfunctional thinking (Garratt et al., 2007). Dysfunctional thoughts can be organized into different levels, some thoughts seem to occur more on a superficial level (negative automatic thoughts), while other thoughts are derived from more deeply integrated dysfunctional mental representations, that can include ﻿rules, expectations, or assumptions (attitudes) and sometimes even originate from early experiences in the childhood (schemas) (Dozois & Beck, 2008). Second, CBT includes procedures aimed at behavioral activation in order to improve deficits in reward processing (Dimidjian et al., 2011), such as a reduced response to reward or an oversensitive response to negative feedback (Chiu & Deldin, 2007; Eshel & Roiser, 2010; Smoski et al., 2009). The third therapy process, the acquisition of CBT skills, is related to both dysfunctional thinking and behavioral activation. CBT skills are defined as the ability to re-evaluate the accuracy of one's own dysfunctional beliefs (CT skills) and in this way change patterns of dysfunctional thinking and the ability to engage proactively in pleasurable activities as a way to target reward experience (BT skills) (Strunk et al., 2007).

The acquisition of CBT skills is maybe one of the most promising therapy processes of CBT for depression. In contrast to the procedures focused on cognitive change and behavioral activation, CBT skill acquisition is a therapy process that emphasizes the patients' ability to use these cognitive change and behavioral activation procedures themselves, outside the therapy sessions. In addition, successful use of CBT skills may protect the patient from developing new future episodes after successful treatments (Strunk et al., 2007). Research shows that after successful treatment, impairments such as dysfunctional mental representations (Arntz, 2020; Sheppard & Teasdale, 2004), negative processing of information (Elgersma et al., 2019; Spinhoven et al., 2018; Woody et al., 2017) or blunted reward processing (Dichter et al., 2012; Pechtel et al., 2013) may remain, thereby possibly increasing the risk of new depressive episodes. The acquisition of CBT skills might be essential to transfer learned content from the therapy session to daily life and to cope with dysfunctional therapy processes or symptoms in future scenarios outside the therapeutic context.

However, although multiple studies have pointed out that the acquisition of CBT skills is associated with reduced depression (Adler et al., 2015; Forand et al., 2018; Strunk et al., 2014; Webb et al., 2019) and seems specific to CBT (Bruijniks et al., 2022), it is still unknown whether CBT skill acquisition directly causes a reduction of symptoms of depression. In order to test the causal effects of a certain therapeutic procedure, it is necessary to isolate the procedure and investigate its direct effects on the hypothesized changes in therapy processes and outcome (Bruijniks et al., 2018). Two preliminary experiments that focused on the acquisition of CT skills already evaluated how a procedure focused on the acquisition of CT skills could be isolated (Bruijniks et al., 2018) and showed that a short cognitive skill acquisition procedure in the form of a group masterclass led to better CT skill acquisition compared to an active control procedure in a sample of distressed students (Bruijniks, Los, & Huibers 2020). A next step towards the clinical application of this finding would be to evaluate how a procedure focused on CBT skill acquisition causally affects the acquisition of CBT skills and subsequent symptom reduction in a clinically depressed sample.

Nevertheless, skill acquisition seems to be a multifaceted process that requires different cognitive and neurobiological resources (Anderson et al., 2016, 2018; Basak et al., 2011; VanLehn, 1996), which may be impaired in depressed patients. Compared to healthy individuals, depressed individuals have biased attention towards negative rather than positive information (Fu et al., 2008; Liu et al., 2012; Marchetti et al., 2018; Roiser et al., 2012) and suffer from a variety of deficits related to executive functioning, such as inhibition, planning and working memory (Snyder, 2013). Recent studies supported the hypothesis that individual differences in cognitive or neurobiological impairments may interfere with the success of psychological treatments. In a systematic review, cognitive and neurobiological impairments showed to be associated with impairments in dysfunctional thinking and reward processing while depressed patients with better cognitive control, but more emotional bias, before start of CBT seemed to benefit more from CBT’s procedures (Bruijniks, DeRubeis, et al., 2019). Possibly, patients who show more emotional bias are better able to tolerate and therefore target emotions as part of the CBT (Stange et al., 2017) while individuals with better cognitive capacities are more capable of integrating and implementing new information that was retrieved in the therapy session. CBT might help individuals with increased emotional bias, but limited cognitive capacity, to regain (emotional) control (Siegle et al., 2006). A recent experiment supports this suggestion, as results indicated that in healthy participants who received a stress induction, executive control under stress, but not under non-stressful circumstances, predicted the ability to reappraise negative material to become less negative (Quinn & Joormann, 2020). Investigating whether individual differences in cognitive or neurobiological impairments are associated with the success of a CBT skill acquisition procedure will provide insight in for whom CBT skill acquisition procedures will be more or less effective.

If individual differences in cognitive or neurobiological impairments are related to the success of CBT skill acquisition, this also means that the success of CBT skill acquisition might be improved by increasing the patients' capacity to learn from these procedures. One way to address and improve cognitive and neurobiological processes during treatment is by providing cognitive support. Examples are the use of memory strategies within sessions of CBT (Harvey et al., 2014, 2017) or providing short retrieval tests between the sessions (Bruijniks, Sijbrandij, et al., 2020). The major hypothesis is that by enhancing recall for the session content, the success of psychotherapy outcomes for depression can be improved and some studies provided preliminary evidence for this hypothesis (Dong, Lee, et al., 2017). However, to improve psychotherapy it might not only be important to improve recall of the session content but also improve and develop the therapy process. Yet, while current cognitive support strategies such as retrieval of newly learned information may improve recall of the session content, it might not be enough to improve CBT skill acquisition. According to theories on skill acquisition, the process of skill acquisition starts with learning new information (this can also be seen as the 'declarative' part of skill acquisition), but then repeated practice is necessary to turn it into a more procedural form in which the newly learned skill becomes more and more automatized over time (Anderson et al., 2018; Tenison & Anderson, 2016; VanLehn, 1996). To increase skill acquisition, it might therefore be necessary to use a strategy that supports both the declarative and procedural parts of memory.

One strategy that seems promising in affecting both declarative and procedural memory is mental imagery. Mental imagery refers to perceptual experiences in the absence of sensory input and constitutes a non-verbal way of information processing, closely related to the experience of emotions (Holmes & Mathews, 2010). Mental imagery allows us to simulate past and future experiences and because of this allows us to “try-out” different courses of actions and their emotional consequences (Ji et al., 2016; Moulton & Kosslyn, 2009). Given these properties of mental imagery, when applied to CBT skills, imagery could be used to simulate skill application (Renner et al., 2021). Mental imagery has been linked to improved acquisition of skills in non-clinical settings, such as tennis performance or the development of surgical skills (Anton et al., 2017; Dana & Gozalzadeh, 2017; Gregg et al., 2011; Kim et al., 2017; Kraeutner et al., 2016), but also to increased BT skills in a clinically depressed population (Renner et al., 2017). Additionally, mental imagery has been related to the improvement of cognitive functioning, such as recall of memories (Dalgleish et al., 2013) and prospective memory (i.e., memorizing to execute a previously formed intention at some point in the future; McFarland & Glisky, 2012; McFarland & Vasterling, 2018). We suggest that simulating applying CBT skills using mental imagery might be a potential efficient way to increase skill acquisition during psychotherapy (Renner et al., 2021).

The aims of this randomized trial are two-fold. The first aim is to investigate and compare the direct effects of three procedures (active control, CBT skill acquisition (CBTSAP), CBTSAP + mental imagery) on changes in therapy processes (the acquisition of CBT skills, changes in idiosyncratic dysfunctional thoughts and behaviors, general dysfunctional thinking and reward processing) and depressive symptoms in a sample of patients with a diagnosis of major depressive disorder who do not currently receive other psychological treatment. We expect that compared to an active control procedure, the procedures focused on CBT skill acquisition (CBTSAP and CBTSAP + mental imagery) will lead to more improvement in the therapy processes and depressive symptoms. Second, we will investigate whether the effect of the therapeutic procedures is moderated by individual differences in learning capacity. Learning capacity will be defined as the presence of memory functioning and emotional bias (i.e., where more emotional bias and better memory functioning are defined as better learning capacity). Following earlier literature on the measurement of memory (Unsworth, 2010; Wilhelm et al., 2013), the measurement of memory functioning will be composed of both working memory and long-term memory tasks. We expect that, compared to the active control procedure, better learning capacity will be associated with larger improvements in the therapy processes and depressive symptoms in both the CBTSAP’s. In addition, we expect a difference between CBTSAP with versus without mental imagery: patients with low learning capacity will have most benefit from mental imagery and lower learning capacity will therefore be associated with more improvement in the therapy processes and depressive symptoms in the CBTSAP with mental imagery compared to the CBTSAP without mental imagery condition.

Besides the two main aims of the study, additional secondary analyses will be conducted. Because earlier studies suggested that cognitive support might improve the effects of therapy by increasing memory of the session content (Dong, Lee, et al., 2017), we additionally included a measure of session recall in the study and will test whether session recall will differ between the procedures. Hypotheses of these secondary analyses are in line with our hypotheses for the main study aims: we expect session recall to be larger in the CBTSAP with mental imagery compared to the CBTSAP without mental imagery. In further secondary analyses we will investigate whether the effect of the procedures on depressive symptoms is mediated through one of the therapy processes and/or session recall and whether these mediation effects are specific to the CBTSAP's (compared to the active control procedure).

**Figure 1:**
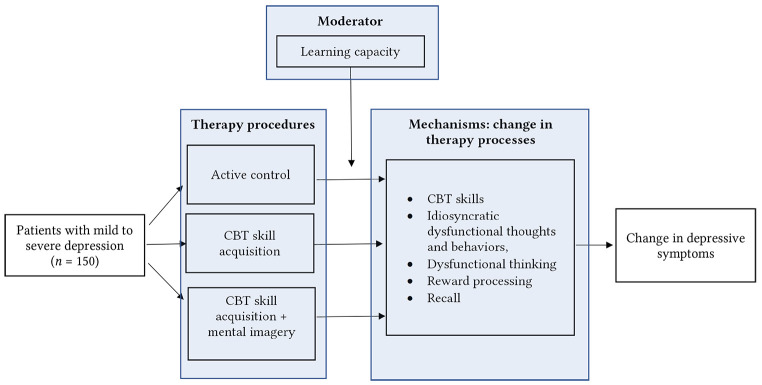
Conceptual Model for the Proposed Study

A conceptual model for the proposed study can be found in Figure 1.

## Method

### Design

Between-subject experimental design with three parallel conditions, each having an equal length of 3 x 45-minute sessions: 1) Active control procedure (*n* = 50), 2) CBT skill acquisition procedure (CBTSAP) (*n* = 50), 3) CBTSAP with mental imagery (*n* = 50). The Ethic Committee of Freiburg University approved the study (registration number: 20-1022) and the trial is pre-registered at the German Clinical Trial Register (DKTR; registration number: DRKS00024116).

### Participants

We aim to include 150 patients with a primary diagnosis of a major depressive disorder (MDD) (excluding non-dysthymic persistent major depressive disorder) as indicated by Structural Clinical Interview for DSM-5 Disorders (SCID-5-CV) and a score on Beck’s Depression Inventory II (BDI-II) ≥ 14 to ensure sufficient symptom severity. Patients should be aged between 18-65 and have sufficient knowledge of the German language (because therapy sessions will be held in German). To prevent any potential interference with the therapeutic procedures and/or measurement of learning capacity, patients with the presence of a previously stated diagnosis of attention-deficit/hyperactivity disorder or attention-deficit disorder, current drug or alcohol use disorder according to the Structural Clinical Interview for DSM-5 (SCID-5-CV) or a cluster A or B personality disorder known by admission to the treatment center are excluded. To ensure the effects are attributable to the current therapeutic procedures, patients who receive currently (other) psychological treatment or have received CBT focusing on a major depressive disorder in the previous year are excluded. To reduce risk of adverse events, patient who show a high risk of suicide according to the intake staff or a score > 1 on BDI_II item 13 (Suicidal thought or wishes) will be excluded.

### Sample Size

Based on a medium effect size, alpha = .05, power =.80, number of experimental conditions = 3, number of repeated measurements = 2 to 4 (G*power (Faul et al., 2007)), a total sample size of 102 to 120 participants would be needed to detect a main effect, and 42 to 57 participants to detect an interaction in a repeated measures ANOVA. Simulation studies suggest 80 to 100 participants to detect an interaction effect between three groups (Shieh, 2019) while simulation studies on multilevel analyses suggest *n* = 80 participants to detect a medium effect size with power =.80 (Aarts et al., 2014). Taking into account 20% drop-out, we aim to include a total of 150 participants.

### Recruitment

Patients will be recruited in two different ways. First, patients will be recruited from the academic outpatient treatment center at the Department of Psychology, Unit for Clinical Psychology and Psychotherapy at the Albert-Ludwigs University of Freiburg. Patients with various mental health problems and with a large variety of socio-demographic backgrounds seek treatment at the clinic. Patients can receive up to 80 sessions individual CBT at the clinic in accordance with the German national health insurance regulations. Treatment seeking individuals with severe mental disorders (e.g. schizophrenia) or acute suicidality are referred to other specialized services outside the clinic or in-patient treatment if indicated. During the intake patients will be checked on in- and exclusion criteria and receive the patient information letter if they are potentially eligible for study participation. After one week, patients will be called to check whether they are interested in participating in the study. The remaining in- and exclusion criteria will be checked, and the Structural Clinical Interview for DSM-5 Clinical Version (SCID-5-CV) interview will be conducted by phone. The procedures will take place while the patient is on a waiting list for regular treatment at the outpatient clinic. Second, individuals can sign up for the study independently of treatment in the academic outpatient treatment center. Information about the study will be put online and distributed in local health care centers. If interested, individuals will be send the patient information letter, called after one week to check remaining in- and exclusion criteria and a SCID-5-CV will be planned.

### Randomization and Procedure

**Figure 2:**
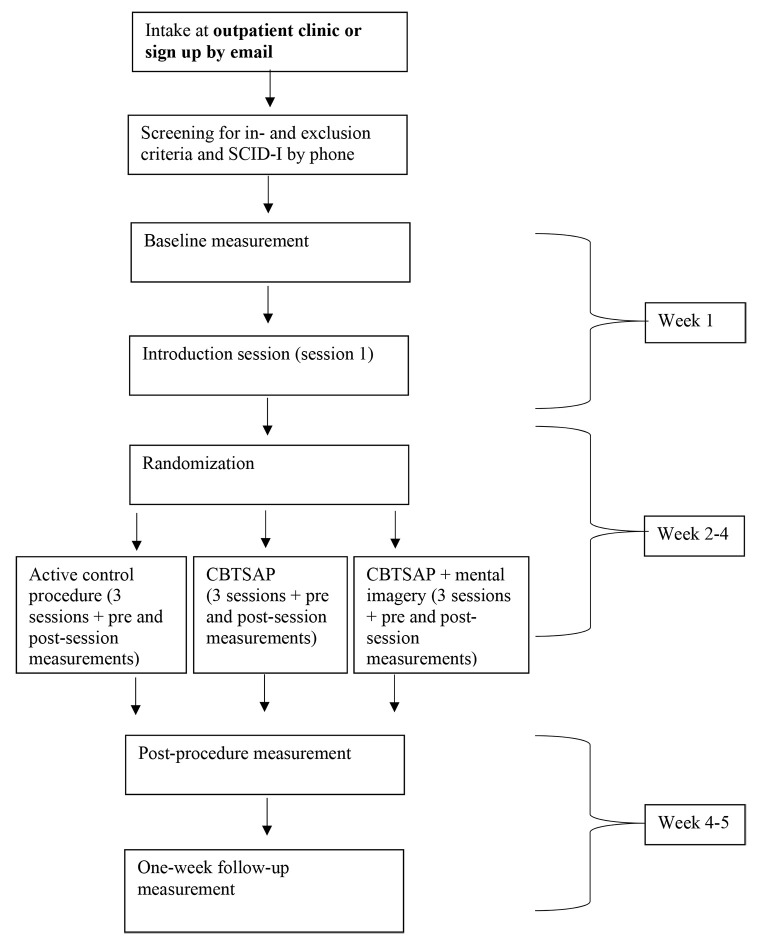
Recruitment and Study Procedure *Note.* The exact time point of each measurement is given in Table 1.

Patient who are eligible to participate in the study will complete a baseline measurement and an introduction session on different days. The baseline measurement will take place in the lab and includes a measurement of learning capacity. Informed consent will be signed before the baseline measurement. The order of the measurements during the baseline measurement will be randomized for each participant in order to control for potential fatigue effects. The introduction session is conducted by the therapist and focuses on introducing the principles of CBT and completing the Core Belief Interview (CBI; McBride et al., 2007). After the introduction session, patients will be randomized into one of the three conditions ﻿using a computer script performing block randomization (1:1:1, block size = 15). Block randomization will be done by a researcher who is not involved in the study measurements. Randomization will be pre-stratified on severity of depression (mild [Beck Depression Inventory-II (BDI-II) = 14-19] vs. moderate to severe [BDI-II ≥ 20]). Therapy sessions will be completed weekly and the total study procedure from baseline measurement to the post measurement will take a maximum of 5 weeks. The researchers who perform the study measurements are blind for the therapeutic procedures. The full study procedure is also presented in Figure 2. Participants do not receive financial incentives for participation in this study.

### Therapists

The therapeutic procedures will be conducted by 5 licensed therapists who are working at the outpatient treatment center of the University of Freiburg. All therapist involved in this study had completed a 3-year fulltime psychotherapist CBT training course required and strictly regulated in Germany to obtain a license as clinical psychological psychotherapist. Therapist have between 5 and 29 years therapy experience. Before start of the study, the therapists received 8 hour training consisting of advanced training in CBT skills by dr. Strunk (Ohio State University), advanced mental imagery training conducted by dr. Renner (University of Freiburg) and elaborate training on the different protocols for each therapeutic procedure in the study. All therapists will be involved in the delivery of all different procedures.

### Introduction Session

The introduction session will focus on introducing the principles of CBT (central focus will be on the relation between thoughts, behaviors and mood) and completing the CBI. During the CBI, the therapist and patient try to gain insight in the current three most relevant dysfunctional beliefs and current three most relevant dysfunctional behaviors for the patient. These beliefs and behaviors will be used in the subsequent sessions to discuss in relation with depressive symptoms (active control procedure) or to practice CBT skills (CBTSAP and CBTSAP + mental imagery condition). ﻿

### Therapeutic Procedures

**Figure 3:**
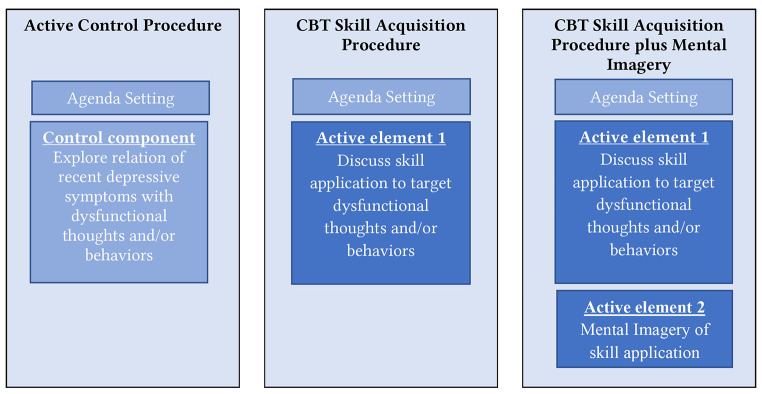
Therapeutic Elements per Procedure *Note.* Detailed information on the session content can be found in data supplement 1 and 2.

All therapeutic procedures use techniques from the protocol for cognitive behavioral therapy (CBT; Beck et al., 1979) for depression and use agenda setting to structure the sessions. In addition, each procedure will focus on targeting the idiosyncratic beliefs and behaviors that were established during the CBI. However, the procedures differ in the number of active ingredients (see Figure 3 and data supplement 1). During the active control procedure, the therapist and patient will focus on discussion of dysfunctional thoughts and behaviors only. Therapists in this condition will be explicitly instructed to focus purely on exploring the relation between dysfunctional thinking and behavior and depressive symptoms, and not to engage in evaluating dysfunctional thinking or behavioral activation. During the CBT skill acquisition procedure (CBTSAP), the therapist and patient will choose one of the cognitive or behavioral skills from a predefined list of CBT skills (i.e., consisting of behavioral activation (behavioral therapy skill) and questions used for evaluating dysfunctional thoughts (cognitive therapy skill). Subsequently, the therapist and patient will discuss how this skill could be or have been applied in past or future situations in which idiosyncratic beliefs and behaviors may (have) lead to negative mood. During the CBTSAP plus mental imagery, the therapist and patient will not only discuss application of the skill but in addition, engage in a mental imagery exercise of skill application. The mental imagery exercise is based on a guided mental imagery procedure and has been shown to increase motivation for goal directed behaviors (Heise et al., 2022; Renner et al., 2019). During the mental imagery procedure, participants are instructed to imagine as vividly as possible and focusing on the positive aspects of the image. The procedure consists of the following steps: 1) imagine the contextual cues (e.g., place, date) of a future or past situation with depressive symptom(s), 2) engaging in multi-sensory imagery of applying the CBT skill in this situation, 3) imaging and experiencing the positive aspects related to successfully applying the CBT skill. All sessions will be videotaped for treatment fidelity checks. Research intervision will take place regularly. The agenda for each therapeutic procedure and the list of CBT skills that can be chosen from and practiced in the CBTSAP procedures is given in data supplement 1 and 2.

### Instruments

An overview of all patient measurements is given in Table 1. An overview of all measurements completed by the therapists or third observers can be found in Table 2.

**Table 1:** Overview of Patient Instruments per Time Point

Measurement instruments	Baseline	Introduction session	Session 1	Session 2	Session 3	One day after Session 3	Follow-up
Primary outcomes: therapy processes							
*CBT skills*							
Ways of Responding (WOR)	X						X
Behavioral Activation for Depression Scale – short form (BADS-SF)	X	X	X	X	X		X
Cognitive Change Sustained Change (CCSC)	X	X	X	X	X		X
*Idiosyncratic thoughts and behaviors*							
Core Belief Interview (CBI)		X	X	X	X	X	X
*Dysfunctional thinking*							
Cognition Checklist (CCL)	X					X	X
*Reward processing*							
Reward Probability Index (RPI)	X					X	X
Temporal Experience of Pleasure (TEPS)	X					X	X
Secondary outcomes: symptoms							
*Depression*							
Beck Depression Inventory II (BDI-II)	X	X	X	X	X		X
*Symptoms other than depression*							
Brief Symptom Inventory (BSI)	X						X
Potential moderators: Learning capacity							
*Memory functioning*							
Verbal working memory: n-back task	X						
Visual working memory: Single probe detection task	X						
Long-term memory Paired-associates task	X						
*Emotional bias*							
Free viewing eye-tracking task	X	X				X	
Other measures							
*Recall*							
Patient Recall Test (PRT)			X	X	X	X	X
*Diagnostics*							
SCID-5-CV	X						
***Demographics***	X						
***Treatment evaluation***							X
***Manipulation check***			X	X	X		
***Procedure check***							X
***Expected success***	X		X				

*Note.* CBTSAP = CBT Skill Acquisition Procedure; MI = Mental Imagery; Structural Clinical Interview for DSM-V Clinical Version (SCID-5-CV).

**Table 2:** Overview of Therapist/Observer Instruments per Time Point

Measurement instruments	Baseline	Introduction session	Session 1	Session 2	Session 3	Post-procedure	Follow-up
Therapy processes							
Therapist-rated recall			X	X	X		
Manipulation check							
Therapy integrity							X
Protocol deviations			X	X	X		

#### Primary Outcome: Therapy Processes

##### CBT Skills

CBT skill acquisition will be measured in two different ways. First, before and one week after the therapeutic procedure patients will complete the Ways of Responding (WOR; Barber & DeRubeis, 1992). During the WOR, CBT skills of the participants are tested by asking them to think about themselves in various situations and to tell what they would think and do in such situations. The WOR will reflect the level of CBT skills demonstrated by the patient. Patients will receive three scenarios before treatment and three different scenarios after treatment. Answers to each scenario will be coded into 25 different categories (more categories per answer possible) and given a rating of the overall quality of the response (i.e., the raters’ judgment on how well the response would be in improving mood or adjusting to the individual’s needs, range = 1 (very negatively) to 7 (very positively)). The total score will be composed of the number of responses on positive categories (responses considered consistent with CBT) minus the number of responses on negative categories (depressotypic statements). Interrater reliability showed to be high (ranging from α = .91 to α = .98) and discriminant and convergent validity have been supported (for example: the WOR showed no correlation with a measure of self-control, but was correlated to self-report measure of CBT skills) (Barber & DeRubeis, 2001; Strunk et al., 2014). Second, change in CBT skills during the procedures will be measured using the Behavioral Activation for Depression Scale – short form (BADS-SF) (BA skills) and Cognitive Change Sustained Change (CCSC) (CT skills). The BADS-SF consists of nine items, each rated on a 7-point Likert Scale and internal consistency (α = .81) and construct and predictive validity were supported (for example: the BADS-SF was positively related to measures of reward, negatively related to measures of avoidance and predicted time spent in high and low rewarding behavior; Manos et al., 2011). An example item from the BADS-SF is: ‘There were certain things I needed to do that I didn’t do’. The CCSC consists of 9 items rated on a 7-point Likert scale and internal consistency was supported (α = .93) and the scale showed convergent and discriminant validity by showing a relation with a self-report scale of CBT skills and no relation with a measure of attributional styles (Schmidt et al., 2019). An example item from the CCSC is: ‘I noticed myself thinking less negatively.’

##### Idiosyncratic Thoughts and Behaviors

Idiosyncratic thoughts and behaviors will be measured with the Core Belief Interview (CBI; McBride et al., 2007). This interview will be completed by the therapist during the introduction session. Together the therapist and patient will form an idiosyncratic top three of dysfunctional thoughts and top three of dysfunctional behaviors. Note that the behaviors can both exist of the presence of unhelpful behaviors or the absence of rewarding behaviors. Based on the identified beliefs and behaviors, six idiosyncratic visual analogue scales (VAS) (0-100) will be constructed for each patient (i.e., three dysfunctional beliefs, three dysfunctional behaviors). For the dysfunctional beliefs, credibility of the beliefs and strength of related emotions will be rated. Presence, reward and pleasure related to the behaviors will be measured. The CBI has been used successfully before to establish idiosyncratic dysfunctional thoughts (Bruijniks, Los, & Huibers, 2020; Renner et al., 2018). The exact items of the CBI are given in data supplement 3.

##### General Dysfunctional Thinking

General dysfunctional thinking will be measured using the Cognition Checklist (CCL; Taylor et al., 1997). The CCL consists of 26 items rated on a 5-point Likert Scale and can be divided into two subscale measuring dysfunctional thoughts related to depression versus anxiety. ﻿Internal consistency (ranging from α = .91 to α = .93) and validity was supported in an outpatient sample (i.e. the depression subscale showed a higher relation to other depression measures compared to the anxiety subscale, and the same was shown in the reverse direction; Steer et al., 1994). An example item of the CCL is: ‘When I am with a friend I think: I’ll never be as good as other people are.’

##### Reward Processing

Reward processing will be measured using the Reward Probability Index (RPI; Carvalho et al., 2011) and the Temporal Experience of Pleasure Scale (TEPS; Gard et al., 2006). The RPI is a 20-item self-report instrument that measures the presence of environmental reward, while the TEPS is an 18-item self-report instrument that measures the ability to experience pleasure. Reliability (RPI: α = .93, TEPS: α = .75) and discriminant and convergent validity have been supported for both instruments (for example: the RPI was related to another measures of reward, but not to measures of anxiety and support, and was related to experiencing rewarding behavior; the TEPS was relatable but also distinguishable from other measures of motivation and pleasure; Carvalho et al., 2011; Gard et al., 2006; Simon et al., 2018). An example item of the RPI is: ‘I have many interests that bring me pleasure.’ (RPI). An example item of the TEPS is: ‘I enjoy taking a deep breath of fresh air when I walk outside’.

#### Secondary Outcome: Psychological Symptoms

##### Depression

Depression will be measured with the Beck Depression Inventory-II (BDI-II; Beck et al., 1996). The BDI-II is a 21-item self-report instrument assessing depressive symptoms during the last two weeks. The items are rated from 0 to 3, higher scores representing more symptom severity. A score 0–13 indicates minimal depression, 14–19 mild depression, 20–28 moderate depression and 29–63 severe depression. Reliability and validity have been supported (i.e., test retest reliability between .73-.96, α = .85, convergent and discriminant validity; Beck et al., 1988; Wang & Gorenstein, 2013). For the purpose of this study, the BDI-II will be adjusted to assess depressive symptoms during the past week.

##### General Psychological Distress

Additional psychological symptoms will be measured using the Brief Symptom Inventory (BSI; (Derogatis & Melisaratos, 1983). The BSI consist of 53 items rated on a 0 (not at all) to 4 (extremely) scale and includes the following subscales: somatization, obsessives-compulsive, interpersonal sensitivity, depression, anxiety, hostility, phobic anxiety, paranoid ideation and psychoticism. Reliability and validity of the scale have been supported (i.e., Cronbach’s alpha of .85, concurrent and divergent validity was supported; de Beurs & Zitman, 2006; Geisheim et al., 2002).

#### Potential Moderators: Learning Capacity

##### Memory Functioning: Verbal Working Memory

Verbal working memory will be measured using the N-back task (Braver et al., 1997). The n-back task measures verbal working memory. During the n-back task participants will be asked if a letter on the screen matches a letter previously (1-back, 2- back, 3-back) presented for 500 ms with an interval of 2000 ms. WM load increases as the task progresses from 1-back to 3-back. Accuracy of responses (total of correct hits (% correctly identified *n*-backs) and correct no hits (% correctly identified no presence of a *n*-back)) are measured and will be used as an outcome measure. The *n*-back task has been considered as a valid measure of working memory (Cronbach’s alpha = .92; Schmiedek et al., 2014; Wilhelm et al., 2013).

##### Memory Functioning: Visual Working Memory

Visual working memory will be measured using the probe change detection task (PCDT; Dai et al., 2019). The PCDT consists of the following steps: 1. Participants are instructed by an arrow on their screen to focus on the left or right side of the screen (200 ms), 2. After a short break (300 ms) the screen is filled with colored squares on a gray background (100 ms). The squares are equally distributed between the left and right side of the screen. Participants are instructed to remember only the squares on the side that was instructed under Step 1, 3. After a second blank screen (900 ms), participants see again a field with colored squares (750 ms) and have to indicate whether the squares on the side of the screen are the same as under Step 2. Set sizes of the trial different between 8 to 12 colored squares in total. Participants will receive a total of 300 trials. Reliability and validity has been supported (i.e., test retest reliability between .52-.75; Dai et al., 2019).

##### Memory Functioning: Long-Term Memory

Long-term memory will be measured using the paired associates task (PAT; Unsworth et al., 2009). Long-term memory can also be considered as 'secondary memory', i.e., the part of memory where information is stored when the primary memory, where new information is temporary maintained is full. During the PAT, participants will be given three lists of 10 non-semantically related word pairs. All words are common nouns, and the word pairs will be presented vertically for 2 sec each. Participants will be told that the cue would always be the word on top and that the target would be on bottom. After the presentation of the last word (which takes 20 seconds), participants will see the cue word and "???" in place of the target word. Participants will be instructed to type in the target word from the current list that matches the cue. Cues will be randomly mixed so that the corresponding target words are not recalled in the same order as that in which they had been presented (i.e., this means that the time between encoding and recall varies and lies between the 2 and 70 seconds). Participants will have 5 sec to type in the corresponding word. A participant’s score is the proportion of items recalled correctly. Words will be taken from the Toronto Word Pool (Friendly et al., 1982). The paired associates task has been considered a valid task of long-term memory (Unsworth et al., 2009; Wilhelm et al., 2013).

##### Emotional Bias: Sustained Selective Attention to Emotional Stimuli

Selective attention will be measured using a free-viewing eye-tracking task (Klawohn et al., 2020). Participants will view two blocks of neutral and happy and neutral and sad faces in counterbalanced order while their gaze patterns are concurrently recorded as index of selective spatial attention. Each block will take 30 trials that last 6 seconds. Each trial will show 16 different faces and participants will be asked to freely view the trials. Participants’ gaze location and duration will be assessed using Eyelink eye-tracker software (https://www.sr-research.com/). The present study will use the exact same task as was recently used and validated by Klawohn and colleagues (2020). To maximize reliability of the task (MacLeod et al., 2019), it will be completed twice at baseline. To further investigate the predictive value of the task, an additional post procedure measurement (i.e., at one week follow-up) will be completed.

#### Other Measures

##### Recall

Patient recall will be measured using the Patient Recall Test (PRT; Lee & Harvey, 2015). The PRT measures recall of the previous session content. Following procedures of Lee & Harvey, the patient will be given 10 minutes to remember as much treatment points from the previous session as possible (past session recall). In addition, cumulative recall (i.e., what is remembered from all sessions) will be measured at the follow-up session. Treatment points will be defined as remembering insights, skills and strategies of the CBT model. Scores will be rated by two independent raters, inconsistencies in scoring will be resolved by discussion. Interrater reliability between raters will be computed. The PRT showed good interrater reliability in previous studies (ICC = .92; Dong, Zhao, et al., 2017). In addition to recall of the patient, therapists are also asked to give a rating of recall on a 1-10 VAS scale (1 = patient has no memory of the previous session, 10 = patient remembers everything perfectly).

##### Manipulation Check

To check if patients in the CBT skill acquisition + mental imagery condition engaged in vivid mental imagery, they will be asked to note how vivid the imagery of the skills practiced in this session was on a scale from 1 (not vivid at all) to 10 (extremely vivid). To check and potentially control for self-efficacy, motivation and anticipated reward in the analyses, participants in all conditions will complete questions on a 0-10 scale and asked to rate based on today's session how capable they feel in coping with their dysfunctional beliefs and behaviors, their motivation to use the content of today's session to do something different in the upcoming week and their anticipated reward of doing something different in the upcoming week based on today's session. In addition, expected success of the SKILT study sessions in reducing depressive symptoms will be asked at baseline and after the first session. At the end of the study, patients will be asked to rate on a 0-10 scale to what degree the received sessions contributed to an improvement in depressive symptoms.

##### Adherence

***Protocol Deviations.*** After each session, the therapist will complete a short questionnaire to check 1. how many skills and application of these skills were discussed, 2. How many mental imagery exercises were conducted, and 3. ask for the presence of deviations to the protocol in that session.

***Procedure Integrity.*** To ensure the procedures differ in the presence of active components (i.e., CBT skill application and use of mental imagery) all sessions will be video-taped. A questionnaire will be developed that measures the presence and duration of the different components in the therapeutic procedures. This questionnaire will be completed by two independent raters.

##### Diagnosis

The SCID-5-CV (First et al., 2019) will be completed by phone during the recruitment phase.

### Data Analyses

All statistical tests will be two-tailed (significance level alpha .05). Descriptives (means, standard deviations) for all measures will be provided for each condition. All analyses will be intention-to-treat and missing data will not be imputed.

#### Main Analyses

The main analyses will be conducted in Stata. First, to test the direct effects of the different procedures on change in therapy processes and symptoms, differences on the primary and secondary outcomes between conditions (CBTSAP versus active control; CBTSAP versus CBTSAP + mental imagery; active control versus CBTSAP + mental imagery) will be tested using multilevel analysis with maximum likelihood estimation (measurements [Level 1] nested within patients [Level 2] nested within therapists [Level 3]). Because the WOR is only measured at two time points, differences between conditions on the WOR will be tested using a repeated measures ANOVA.

Second, to test whether the effect of the procedures is moderated by individual differences in learning capacity, learning capacity will be added as a moderator in the model. Moderation will be tested by adding learning capacity as a main factor and the interaction between learning capacity and condition to the multilevel regression model. For the WOR, the interaction will be added to the repeated measures ANOVA. Moderation of learning capacity will be tested separately for memory functioning and emotional bias. Memory functioning will be tested separately for each component of memory functioning (i.e., verbal working memory, visual working memory, long-term memory), while controlling for type I error (*p* < .016).

#### Mediation Analyses

The potential role of therapy processes and session recall as mechanisms of change will be tested by testing mediation within latent difference score (LDS) models. In separate LDS models (i.e., a different model for each mediator), we will test the relation of the procedure (CBTSAP's versus active control) on subsequent change in the mediator (i.e., therapy processes: CBT skills, idiosyncratic dysfunctional thinking and behaviors, general dysfunctional thinking, reward processing, and session recall) on subsequent change in the outcome (depressive symptoms). Note that we will merge the two CBTSAP's to test mediation of the active control versus the CBTSAP's. LDS models allow tests of mediation, include the temporality of the effects and are therefore capable of testing potential reverse causality (Grimm et al., 2017; McArdle, 2009).

## Discussion

We presented a protocol for a randomized controlled study that isolates an often-used therapeutic procedure focused on the acquisition of cognitive behavioral therapy skills (CBTSAP) to test its causal effects on psychotherapy outcomes. The CBTSAP will be compared to an active control condition and CBTSAP with mental imagery. We hypothesized that, compared to an active control procedure, the CBTSAP's would lead to direct improvement in CBT skills, related therapy processes (dysfunctional thinking and reward processing) and subsequent reduction of depressive symptoms. In addition, we suggested that individual differences in cognitive and neurological impairments (referred to as learning capacity) in depressed patients may interfere with the successful acquisition of CBT skills and that especially the patients with low learning capacity will benefit from added mental imagery to the CBTSAP.

One major strength of the present study is that it will be the first to investigate the direct effects of an isolated procedure focused on the acquisition of CBT skills in a depressed sample. Although the potential of investigating isolated procedures has been recognized (Craske, 2016; MacLeod & Grafton, 2016; Teasdale & Fennell, 1982), experimental studies that isolate therapeutic procedures in the field of depression have been scarce so far (Bruijniks et al., 2018). In addition, by performing an experiment that informs us about which therapeutic procedure works best for whom, the proposed study taps into the field of personalized medicine (i.e., optimizing the effects of treatment by matching the treatment to the patient (Cohen & DeRubeis, 2018)). By increasing insight in the direct effects of therapeutic procedures on how and for whom they reduce depression, the present study will not only contribute to the research field of personalized medicine, but has the potential to inform and improve clinical practice (i.e., informing on what technique might be helpful for whom).

This study is also the first that elaborately assesses learning capacity at baseline to investigate the moderating role of learning capacity on the effects of isolated CBT procedures. Earlier studies have indicated that depressed patients with more emotional bias and more memory functioning might show better improvement during CBT (Bruijniks, DeRubeis, et al., 2019), but these studies mostly investigated the role of learning capacity on the complete treatment package (i.e., a full CBT that includes multiple CBT procedures), primarily used neurobiological measures or where conducted in an elderly depressed population. The present study will be able to inform on the specific role of learning capacity in a key therapeutic procedure, the acquisition of CBT skills, in CBT for depression. In addition, a better understanding of the role of learning capacity and a CBT skill acquisition procedure on CBT outcomes might open up new avenues for future research on the role of skill acquisition and learning capacity in psychotherapy for depression in general. Another strength of the study is the repeated measurement of therapy processes, which will allow us to investigate how learning capacity affects the hypothesized mechanisms underlying the success of a CBT skill acquisition procedure and also how the CBTSAP might lead to reduction of depressive symptoms through these mechanisms. A final strength of the present study is that it includes a multimodal assessment, using not only self-report instruments but also a CBT skill test, idiosyncratic measures of therapy process change, behavioral tasks and eye-tracking. A limitation of the present study is that it is powered to find medium to large effects and will be underpowered to find small effects between the three treatment conditions.

In conclusion, while there are a number of effective evidenced based treatments for depression, many patients do not improve in treatment and progress in treatment innovation has been slow. One way forward is to isolate specific therapeutic procedures and test their direct effects on therapy processes and outcomes. Based on this experimental research framework we will conduct a randomized clinical study testing the direct effects of a key CBT procedure, CBT-skills, with or without a cognitive support strategy compared to an active control condition. The results of this study will contribute to a better understanding of individual differences in the effects of key CBT procedures.

## Supplementary Materials

The Supplementary Materials contain more exact information on the different therapeutic procedures and the outcomes of the core belief interview as these are used in the study (for access see Index of Supplementary Materials below).



BruijniksS. J. E.
FrankU.
Tuschen-CaffierB.
WerthmannJ.
RennerF.
 (2023). Supplementary materials to "Skill Improvement Through Learning in Therapy (SKILT): A study protocol for a randomized trial testing the direct effects of cognitive behavioral therapy skill acquisition and role of learning capacity in depression"
[Additional information]. PsychOpen. 10.23668/psycharchives.12574
PMC1010315737065002

## Data Availability

Data sharing is not applicable to this article as no datasets were generated or analyzed during the current study.

## Appendix: List of Abbreviations

BADS-SF – Behavioral Activation for Depression Scale – short form
BSI – Brief Symptom Inventory
CBI – Core Belief Interview
CBT – cognitive behavioral therapy
CBTSAP – cognitive behavioral therapy skill acquisition procedure
CCSC – Cognitive Change Sustained Change
CCL – Cognition Checklist
PAT – Paired associates task
PCDT – Probe change detection task
PRT – Patient Recall Test
RPI – Reward Probability Index
LDS – Latent difference scores
SCID-5-CV – Structural Clinical Interview for DSM-V
TEPS – Temporal Experience of Pleasure Scale
VAS – Visual analogue scale
WOR – Ways of Responding
